# The effects of varying gravito-inertial stressors on grip strength and hemodynamic responses in men and women

**DOI:** 10.1007/s00421-019-04084-y

**Published:** 2019-02-07

**Authors:** Olivier White, Marie Barbiero, Nandu Goswami

**Affiliations:** 1 INSERM UMR1093-CAPS, Université Bourgogne Franche-Comté, UFR des Sciences du Sport, 21000 Dijon, France; 2 Otto Loewi Research Center for Vascular Biology, Immunology and Inflammation, Physiology Division, Research Unit: “Gravitational Physiology Aging and Medicine”, Medical University of Graz, Neue Stiftingtalstrasse 6, D-5, 8036 Graz, Austria

**Keywords:** Artificial gravity, Sex, Motor control, Blood pressure, Heart rate, Presyncope

## Abstract

**Purpose:**

The body behaves as a global system with many interconnected subsystems. While the effects of a gravitational change on body responses have been extensively studied in isolation, we are not aware of any study that has examined these two types of body responses concurrently. Here, we examined how the cognitive and cardiovascular systems respond during application of varying gravito-inertial stressors in men and women.

**Methods:**

Ten men and nine women underwent three 5-min centrifugation sessions (2.4 g at the feet, 1.5 g at the heart) in which participants rhythmically moved a hand-held object for 20 s. Grip force and hemodynamic responses were continuously measured during centrifugation and rest periods.

**Result:**

Men optimized the modulation between grip force and the destabilizing load force, but not women. Exposure to artificial gravity induced higher heart rate and mean arterial pressure in both sexes compared to baseline. However, during artificial gravity exposure, only women decreased heart rate across sessions. Interestingly, we found that finishers of the protocol (mostly men) and Non-finishers (mostly women) exhibited divergent patterns of hemodynamic responses.

**Conclusion:**

We speculate that the lack of grip force adaptation reported in women could be linked to the challenged hemodynamic responses during artificial gravity. By deriving a simple model to predict failure to complete the protocol, we found that mean arterial pressure—and not sex of the participant—was the most relevant factor. As artificial gravity is being proposed as a countermeasure in long-term manned missions, the observed effects in grip force adaptation and hemodynamic responses during varying gravito-inertial stressors application are particularly important.

## Introduction

### Spaceflight, physiological deconditioning and orthostatic intolerance

Orthostatic intolerance refers to the inability of a person to maintain upright posture without syncope, a transient loss of consciousness due to inadequate oxygen delivery to the brain (Hinghofer-Szalkay et al. 2011). Orthostatic intolerance remains a problem upon return to Earth from the microgravity environment of spaceflight (Goswami et al. 2013; Blaber et al. 2013). Almost every astronaut returning from space exhibits symptoms of cardiovascular deconditioning (Blaber et al. 2011). However, there is a wide range of susceptibility to orthostatic intolerance after spaceflight, with some astronauts experiencing severe symptoms while others are minimally affected.

The cardiovascular system responds rapidly to changes of the gravitational vector within the body (Goswami et al. 2013). On terrestrial environments, changing posture from lying position to standing up and lying down again imparts varying gravitational loading on the body. In spaceflight, loss of these constant shifts in the body’s gravitational orientation leads to cardiovascular deadaptation that is evident in deterioration of reflexes that maintain blood pressure.

### Artificial gravity: effects and applications

Artificial gravity (AG) administered during spaceflight or in ground-based analogues of spaceflight may prevent deconditioning in different physiological systems as well as prevent the development of orthostatic intolerance upon return to Earth (Evans et al. 2018). Current evidence from studies of ambulatory and deconditioned men and women indicate that 90 min of an individualized short-arm centrifuge AG profile significantly increases participants’ orthostatic tolerance limits via increased blood pressure and cerebral blood flow (Goswami et al. 2015a, b; Evans et al. 2015). However, men and women respond differently to these stressors (Harm et al. 2001). Overall, individualized AG seems to be a good countermeasure against orthostatic intolerance.

In addition to its effect on cortical blood flow, AG also modifies cognitive mechanisms (White et al. 2016) thus challenging the maintenance of optimal motor and cognitive skills. Using resting state fMRI and EEG, AG has been shown to alter cerebellar, sensorimotor and vestibular brain regions (Indovina et al. 2005; Cheron et al. 2014; Cebolla et al. 2016; Rousseau et al. 2016; Van Ombergen et al. 2017). Furthermore, the relationship between brain cortical activity and brain oxygenation in the prefrontal cortex during hypergravity exposure has also been studied (Smith et al. 2013). Specifically, sex-specific changes in cortical activation patterns during exposure to AG have also been reported using EEG (Schneider et al. 2014). It was shown that alpha brain activity increased more in men than in women subject to the same AG. Usually, a decrease of alpha brain activity (and a parallel increase in beta brain activity) is associated with stress and brain arousal (Bonnet and Arand 2001). This suggests that men and women react cognitively differently to AG as well and that this difference may impact the way each sex performs fine motor tasks.

Addressing questions such as how AG affects the body holistically has been marked as a priority research area (White et al. 2016). Here, we investigate how cardiovascular responses interact with the execution of a motor task in new gravitoinertial contexts. The participants cyclically moved an instrumented object along the long body axis aligned with the gravitoinertial direction while their cardiovascular responses were continuously monitored. Assessed specifically was how efficiently participants performed this simple dextrous task during rotation in a short-arm human centrifuge and what the effects were on the hemodynamic responses. In addition, as most women participants could not complete the administered AG protocol, we also identified the profile of participants as Non-finishers (participants in whom at least one of the pre-defined criteria for presyncope was fulfilled) or Finishers (participants who completed the entire protocol), taking into account cardiovascular responses and motor function parameters. Presyncope is characterized by a sudden drop in systolic blood pressure to below 80 mmHg or a drop in heart rate by 15 beats per minute (bpm) or development of symptoms like dizziness, light-headedness, sweating, and ultimately, in transient loss of consciousness (elaborated in Gao et al. 2008; Grasser et al. 2009; Goswami et al. 2011; Hinghofer-Szalkay et al. 2011).

## Materials and methods

### Sample size and participant recruitment

Exposure to whole body centrifugation is demanding and the number of enrolled participants is often constrained. Therefore, we made a power calculation for an error probability (*α*) of 0.05, a power (1 − *β*) of 0.80 and an average effect size (*d*) of 0.5. To achieve statistical significance, this analysis yielded an estimated number of participants equal to 12. This value is compatible with previous studies in which cognitive functions and hemodynamic parameters were assessed during varying gravitational loading (White et al. 2005, 2012, 2018; Smith et al. 2013; Schneider et al. 2014; Goswami et al. 2015a, b; Evans et al. 2015; Barbiero et al. 2017; Verma et al. 2018) (*N* = 6 to 20). We nevertheless decided to apply a safety margin to these values and recruited 19 participants.

Each participant received a comprehensive medical examination from MEDES (French Institute for Space Medicine and Physiology at Toulouse, France), where the study was conducted, prior to participation. We included in the experiment women and men participants aged between 20 and 40 years, with a BMI < 30 kg/m², a normal clinical examination, a normal electrocardiogram and a normal arterial pressure. Participants who have taken part previously in hypergravity experiments in a short-arm human centrifuge and who failed the medical exam were excluded from the protocol. Ten right-handed healthy men (29.7 ± 5.6 years old, 177.8 ± 4.4 cm, BMI 25.1 ± 2.0 kg/m^2^) and nine women (27.6 ± 4.6 years old, 165.1 ± 4.8 cm, BMI 21.9 ± 1.9 kg/m^2^) took part in this protocol.

The study was conducted in accordance with the ethical practices stipulated in the Declaration of Helsinki (1964). Ethics approval was obtained by MEDES (2014-A00212-45). All participants signed the informed consent form, which is stored at MEDES.

### Experimental procedures

**Fig. 1:**
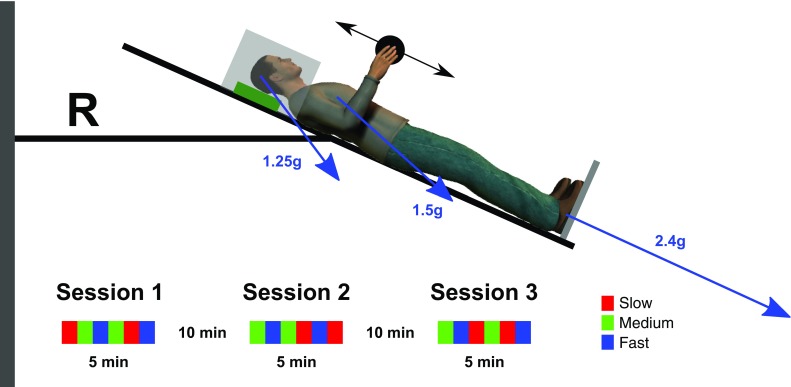
Scaled sketch of the participant in the centrifuge. The leftward thin vertical rectangle represents the axis of rotation of the centrifuge (angular velocity of 2*πT*). The centrifuge nacelle was tilted by 24° and positioned such that the elbow joint was at distance *R* = 1.63 m from the axis of rotation. The participant was supine on the centrifuge nacelle, her/his head resting on a cushion (rectangle) and the feet supported by a metallic plate (line under the feet). Constant contact between the participant and the operator was maintained through headphones. The nacelle had local dark environments to eliminate the influence of external visual stimuli (transparent rectangle). Gravitoinertial vectors depend both on Earth gravity and centripetal acceleration and vary in magnitude and direction (arrows, Head: 1.25 g, − 53°; Heart: 1.5 g, − 42°; Feet: 2.4 g, − 24.6°). The two-headed arrow represents the trajectory of the object (black disk) in the sagittal plane. The lower inset illustrates a complete experimental schedule composed by three sessions. Each color corresponds to a different movement pace condition (see legend)

Each participant was placed in a supine position on the centrifuge (Fig. 1). The participant rested his/her head on a thin pillow with the feet supported by a rigid metallic platform. The participant was equipped with headphones to maintain contact with the operator in the control room. An opaque ventilated box above the head prevented visual feedback of the environment.

### Experimental protocol

We measured how efficiently participants performed a motor task with the right hand (through grip force) and monitored the accompanying hemodynamic responses (heart rate and arterial pressure). The experiment could be interrupted at any time upon participants’ request or by the medical supervisor if presyncopal signs and symptoms developed. The experiment was actually interrupted during the last session (see Experimental procedures) for five women who showed signs of motion sickness and/or developed presyncope (Table 1). The signs for motion sickness included intermittent performance of the motor activity or the participant verbally reporting so.

**Table 1:** Participant details grouped by sex of the participant (F or M)

Participant	Sex	N sessions	Exp. duration (s)	Phys variables available	Finisher
P01	*F*	*2*	*202*	*No*	*No*
P04	*F*	*3*	*592*	*Yes*	*Yes*
P06	*F*	*3*	*857*	*No*	*Yes*
P10	*F*	*3*	*829*	*No*	*Yes*
P11	*F*	*1*	*260*	*Yes*	*No*
P12	*F*	*2*	*100*	*Yes*	*No*
P13	*F*	*3*	*605*	*Yes*	*Yes*
P14	*F*	*2*	*295*	*Yes*	*No*
P18	*F*	*1*	*83*	*Yes*	*No*
P02	M	3	710	Yes	Yes
P03	M	3	767	No	Yes
P05	M	3	525	Yes	Yes
P07	M	3	865	No	Yes
P08	M	3	434	Yes	Yes
P09	M	2	501	No	No
P15	M	3	645	Yes	Yes
P16	M	3	873	Yes	Yes
P17	M	3	342	Yes	Yes
P19	M	3	528	Yes	Yes

The 3rd column (N Sessions) provides the number of completed sessions. The 4th column reports the total duration spent in the rotating centrifuge. In theory, the centrifuge rotated for 900 seconds (3 × 5 min, see details in the "Materials and methods" section). The 5th column indicates if physiological data (HR and MAP) could be analyzed. Finishers (reached the end of the experiment) and Non-finishers (did not reach the end of the experiment or had to skip some conditions) are categorized in the last column

### AG protocol

Participants underwent three centrifugation sessions each lasting for five minutes with the head tilted to 24 degrees upward (Fig. 1). This configuration was adopted to match technical requirements in a complementary study (Barbiero et al. 2017). Each session was separated by 10-min breaks during which the centrifuge nacelle was brought back to supine position and each participant rested quietly in the horizontal position. Participants received 2.4 g at the feet, which approximates to 1.5 g at the heart and 1.3 g at the head. Figure 1 describes the mechanical configuration and the gravitoinertial vectors used in this study.

### Object manipulation task

During centrifugation, and following a signal from the operator, each participant performed rhythmic upper arm movements in the sagittal plane with an instrumented object held between the index and thumb of the right hand. The device recorded the 3d forces applied by the index and thumb finger as well as 3d accelerations of the object (detailed in Barbiero et al. 2017). Movement pace was provided by a metronome controlled by the operator that emitted 2 auditory signals per cycle, one at the top and one at the bottom of the trajectory. The signal was routed via headphones to the participant’s ears. Three paces (Slow = 0.7 Hz, Medium = 1 Hz and Fast = 1.3 Hz) were presented twice each for 30 s (3 paces × 2 repetitions × 30 s = 3 min). Pauses of about 20 s separated movement conditions to prevent fatigue. Pace order was randomized and counterbalanced across participants. The design ensured that participants were comparable across sessions in terms of total mechanical work performed. At the end of each session, the nacelle went back to idle position. All signals were continuously sampled at 200 Hz and stored on a computer laptop strapped on the centrifuge.

### Hemodynamic data recording

Continuous heart rate and arterial pressure were monitored using non-invasive photoplethysmography (Portapres: FMS, the Netherlands). The Portapres finger cuff was placed on the resting left hand during the task. During tilting, it was ensured that finger measuring the blood pressure was held at the level of the heart using a Velcro strap.

### Data collection

Since all participants did not complete the experiment, we recorded the individual total exposure to AG. In addition, participants were categorized according to whether they could complete at least a few movement cycles in each condition and each session (Finishers) or not (Non-finishers). All Non-finishers completed 1 or 2 sessions.

### Data processing and analysis

We continuously recorded object kinematics (3d-acceleration) and the forces applied by the hand on the object (mass, m). These forces are used to characterize how participants manipulate the object. Let us consider first an object held stationery between the thumb and index fingers. In that case, the only force the fingers need to counteract is the object’s weight, W = *m* × *g* (g being gravitational acceleration). This force, that tends to make the object slip out of the grasp, is called the load force. The brain decides to produce a certain magnitude of grip force orthogonal to the object’s surface. If the object is moved, its acceleration (*a*) will add an inertial term (*m* × *a*) that will also need to be taken into account by the brain in order to adjust the most appropriate grip force in real-time. Cognition is involved in this motor to anticipate the variation of load force (now LF = *m* × *g* + *m* × *a*) in function of mass, acceleration but also of variable gravity. We calculated mean grip force during each oscillation phase. Force signals were smoothed with a zero phase-lag autoregressive filter (cutoff 10 Hz). A trial was defined as a series of cyclic movements. On average, per trial, participants performed 19.5 cycles for 0.7 Hz (SD = 6.9), 20.9 cycles for 1 Hz (SD = 2.2) and 26.3 cycles for 1.3 Hz (SD = 2.5).

For each session, we stored average heart rate (HR) and mean arterial pressure (MAP) values for 10-s epochs at baseline (before the centrifuge started), early after the centrifuge started (once HR was stabilized) and late (before the centrifuge ramped down to idle position). Furthermore, to quantify how HR and MAP varied over time, we fitted a linear function through these time series and analyzed the slopes of the regression lines.

### Statistical analysis

Matlab (The Mathworks, Chicago, IL) was used for data processing and statistical analyses. The force and physiological variables were averaged per participant and condition. Data were normally distributed as tested by the Shapiro–Wilk test (force: all *p* > 0.632; physiological: *p* > 0.05). We grouped the data according to sex of the participants (men and women).

We used a Fast Fourier Transform to extract the main frequency component of the acceleration profile for each trial. For each group, we used a two-way repeated measures ANOVA with a within-participant factor session (1st, 2nd or 3rd) and frequency (0.7 Hz, 1 Hz, 1.3 Hz). Post hoc tests were performed with Scheffé tests. Differences between groups were assessed with independent two-tailed *t* tests. We report here partial eta-squared values for significant results (*p* < 0.05, corrected for multiple comparisons with Greenhouse-Geisser) to provide indication on effect sizes.

## Results

Nineteen healthy participants cyclically moved an instrumented object while exposed to AG. We measured two types of body responses simultaneously to assess whether a fine motor action interacts with cardiovascular responses and observe whether sex of the participant modulates these responses in the challenging situations.

### Sex differences in the dynamics of prehension

Participants adopted a pace that approached the instructions. Independent t-tests revealed no difference between actual and theoretical paces of 1 Hz (*t*_24_ = 1.6, *p* = 0.133) but faster paces than instructed in the slowest condition (*t*_24_ = 2.3, *p* = 0.034, ${\eta}^{2}_{\text{p}}$ = 0.17) and slower paces than prescribed in the fastest condition (*t*_24_ = 2.1, *p* = 0.045, ${\eta}^{2}_{\text{p}}$ = 0.16). The ANOVA revealed significant effects of instructed frequency on pace (*F*_2,22_=69.7, *p* < 0.001, ${\eta}^{2}_{\text{p}}$ = 0.86) and mean grip force (*F*_2,22_=11.4, *p* < 0.001, ${\eta}^{2}_{\text{p}}$ = 0.51).

**Fig. 2:**
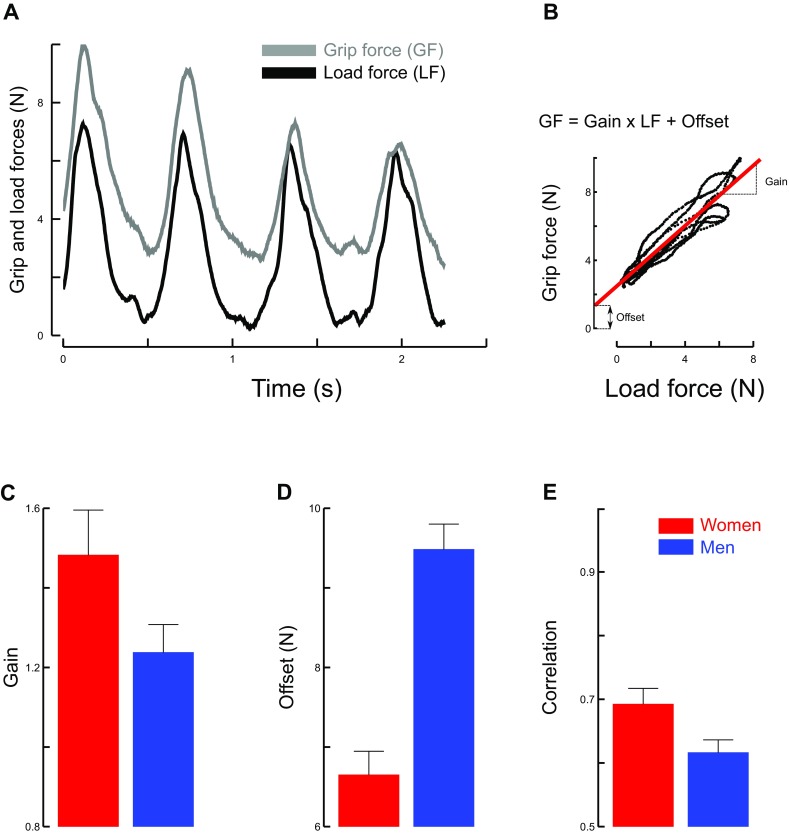
Main variables quantifying the grip force (GF) and load force (LF) synergy. Grip force (gray line) parallels load force (black line) when a mass is moved cyclically along the vertical gravitational axis. **a** Depicts four cycles of movement over time. **b** Highlights the very good linear correlation between these GF and LF (regression line). The “Gain” and the “Offset” quantify this correlation. Average values of gains (**c**), offsets (**d**) and reliability of the linear relationship (**e**) plotted separately in women (red) and men (blue). Error bars represent SEM

Men exerted overall 40.7% larger grip forces than women (*t*_17_ = 2.5, *p* = 0.024,${\eta}^{2}_{\text{p}}$ = 0.27). The brain anticipates the way load forces vary. The hand then exerts the most appropriate grip forces in real time. In other words, when load forces increase or decrease, grip force will vary in the same direction. The efficiency of grip force adjustments is usually measured by computing the correlation between load force and grip force (Flanagan et al. 1993; Flanagan and Wing 1995; White 2015). These correlations reach very high values reflecting anticipatory strategies. This process is illustrated in Fig. 2a, b. The slope of the linear fit between load and grip forces quantifies the grip force increment (*y* axis) for a given load force increment (*x* axis) and the offset captures a safety margin, i.e. the force that would have been applied when load force LF = 0 (see Fig. 2a, b). Men had significantly larger offsets than women (Fig. 2d, *t*_17_ = 2.3, *p* = 0.036, ${\eta}^{2}_{\text{p}}$ = 0.23) but gains (slope of the linear fit) and the quality of the fit (correlation), good on average (*r* = 0.63), were similar (Fig. 2c, gain: *t*_17_ = 0.4, *p* = 0.694 and Fig. 2e, correlation: *t*_17_ = 1.2, *p* = 0.237). When each group was analyzed separately, the ANOVA only showed a significant effect of frequency in men on the offset (*F*_2,6_ =10.3, *p* = 0.031, ${\eta}^{2}_{\text{p}}$ = 0.77) and on correlation (*F*_2,6_ =10.6, *p* = 0.003, ${\eta}^{2}_{\text{p}}$ = 0.64). In both cases, a Scheffé post hoc shows that these variables increase with frequency but only in sessions 1 and 2 (offset: *p* < 0.038; correlation: *p* < 0.017). To sum up, our data demonstrate that the offset (safety margin) and the correlation between grip force and load force (grip synergy) improve in men between sessions 1 and 2 but remain stable in women.

### Sex differences in heart rate and mean arterial pressure responses

**Fig. 3:**
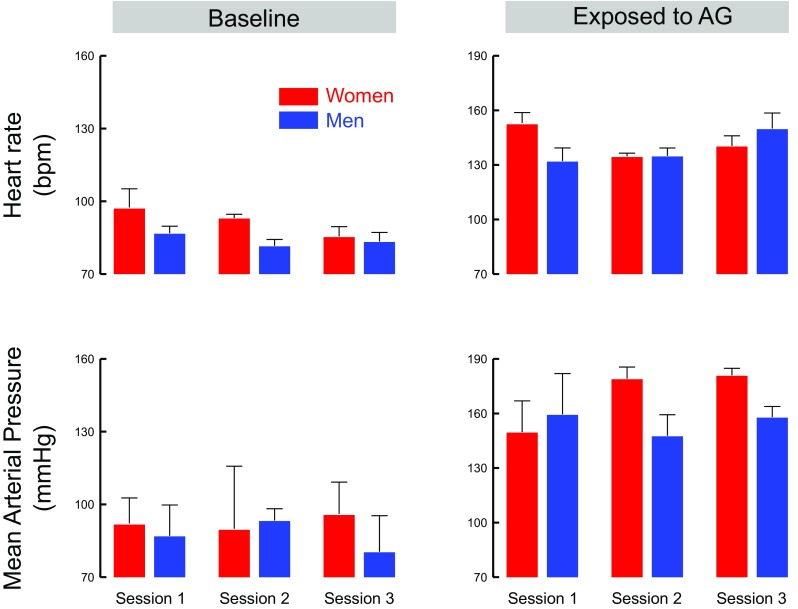
Heart rate (top row) and mean arterial pressure (lower row) at baseline (left column) and during exposure to AG (right column). Data are presented separately in women (red bars) and men participants (blue bars) and across sessions (*x*-axis). Error bars represent SEM

Figure 3 depicts heart rate (HR, upper row) and mean arterial pressure (MAP, lower row) across sessions (1, 2 or 3) and separately in men (black bars) and women participants (gray bars). These variables are plotted separately at baseline (left column) and during the exposure to AG (right column). All participants increased their HR and MAP between baseline and AG exposure (HR: compare upper panels in Fig. 3, *t*_12_ = 20.1, *p* < 0.001, ${\eta}^{2}_{\text{p}}$ = 0.97; MAP: compare lower panels in Fig. 3, *t*_12_ = 11.6, *p* < 0.001,${\eta}^{2}_{\text{p}}$ = 0.92). At baseline, the ANOVA did not show any significant effect of session on HR (Men: *F*_2,8_ = 0.4, *p* = 0.653; Women: *F*_2,2_ = 7, *p* = 0.229) or MAP (Men: *F*_2,8_ = 0.2, *p* = 0.657; Women: *F*_2,2_ = 0.6, *p* = 0.594). However, during AG exposure, women participants decreased their HR across sessions (*F*_2,2_ = 34.6, *p* = 0.028, ${\eta}^{2}_{\text{p}}$ = 0.97, Scheffé post hoc highlights a difference between sessions 1 and 2 only, *p* = 0.014). Men participants remained stable across the sessions (*F*_2,8_ = 1.1, *p* = 0.384). No effect of session was found in MAP during AG exposure across sex (all *F* < 1.8, all *p* > 0.507).

**Fig. 4:**
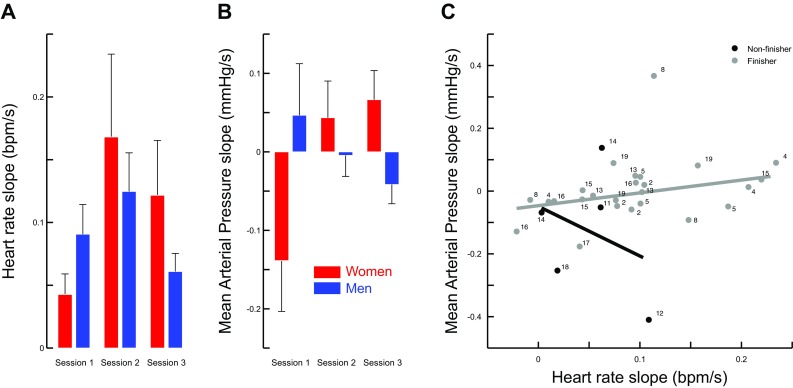
Variation of heart rate (**a**) and mean arterial pressure (**b**) over time, quantified by the slope of the linear regression between these variables and time. Men (blue) and women (red) are separated and slopes are presented for each session. **c** Correlation between HR slope (*x* axis) and MAP slope (*y* axis) in the Non-finishers (black disks) and in the Finishers (gray disks). Labels correspond to participant number

Each session lasted five minutes. We quantified the increasing or decreasing trend of HR and MAP time series by calculating a linear fit (between HR and time or MAP and time). We observed that the slope of HR (Fig. 4a) was not affected by sex of the participant (*t*_11_ = 0.787, *p* = 0.448) or sessions (all *F* < 2.4, *p* > 0.296). However, slopes were all positive, meaning that HR increased during AG exposure (*t*_24_ = 6.1, *p* < 0.001, ${\eta}^{2}_{\text{p}}$ = 0.61). Similarly, the ANOVA did not show any significant effect of *sex* of participant or *session* (main or interaction) on MAP slopes (Fig. 4b, all *F* < 1.1, *p* > 0.489).

Finishers (*N* = 13, 69% men) and Non-finishers (*N* = 6, 83% women) depicted contrasting patterns of HR and MAP. To quantify this relationship, therefore, we calculated HR and MAP relative to baseline and correlated these values separately in Finishers and Non-finishers. Figure 4c illustrates a significant correlation in Finishers (*r* = 0.47, *p* = 0.021), but not in Non-finishers (*r* = − 0.32, *p* > 0.05). Altogether, this analysis shows that HR parallels MAP in Finishers (mostly men) while HR increases and MAP decreases in Non-finishers (mostly women).

## Discussion

Our investigation aimed at identifying interactions between the cardiovascular and cognitive systems between the sexes while participants were exposed to AG. Participants performed oscillatory movements with a hand-held load at different paces with the right hand. We observed that grip force was well adjusted overall and was optimized over sessions. Men exhibited larger safety margins than women. During AG exposure, unlike men, women decreased heart rate across sessions. There was no such effect found in mean arterial pressure. Finally, HR and MAP increased in parallel in Finishers but showed no significant patterns in Non-finishers, therefore yielding an index contrasting these two groups.

The slope of the linear fit between load and grip forces quantifies the grip force increment for a given load force increment (Fig. 2a, b). The larger the slope, the more efficient the grip adjustment. The offset of the fit reflects a safety margin that includes environmental factors and possibly anxiety although its effects on grip force control remains to be demonstrated (Wagner et al. 2015; Rodriguez-Paras et al. 2018). For instance, this margin is slightly larger for a cup of coffee held stationary in an aircraft subject to unpredictable turbulences compared to the same object held in a quiet office. Overall, women exhibited significantly smaller offsets than men which reflects the spontaneous larger forces deployed in men. Both sexes had larger safety margins than in familiar contexts which may be the sign of higher uncertainties during AG exposure. Complex object manipulation tasks performed in new environements take time to adjust, which often initially translates in low gains, large offsets and poor correlations between grip and load forces (Augurelle et al. 2003; White 2015a, b). We observed significant effects of pace on the offset and on correlation but only in men. In both cases, these variables increased with frequency during sessions 1 and 2. This is a signature of an optimization of the motor task early in the experiment and for the two slowest frequencies. It is to be noted that the medium frequency of 1 Hz acted as an attractor for the slowest (uncomfortable) and fastest (too demanding) paces. Indeed, participants could not exactly match the prescribed frequency. Previous work showed that the gravito-inertial context strongly modulates natural movement frequency to optimize energy (White et al. 2008). Collectively, these data demonstrate that the grip synergy is preserved in all conditions but significantly improves over sessions only in men.

### What determines whether a participant will be a finisher during an AG session while doing complex tasks?

To our knowledge, no study has addressed aspects related to completion of motor tasks during centrifugation. As women had difficulties to complete the experiment, and Non-finishers experienced AG a shorter amount of time than Finishers (*t*_17_ = 5, *p* < 0.001, ${\eta}^{2}_{\text{p}}$ = 0.6), we further assessed whether sex of the participant could be used to predict who would complete the motor tasks during centrifugation. However, splitting the data according to sex of participant did not show significant results (*t*_17_ = 1.7, *p* = 0.103), thus suggesting that sex alone is not a predictor of who could complete the motor tasks during AG.

From the above, it appeared that more parameters than sex of the participant needed to be included in the model. Therefore, we carried out a principal component analysis separately in Finishers and Non-finishers, by including the following variables in the analysis: BMI, age, grip force offset, mean HR and mean MAP during AG exposure. We observed that HR and MAP responses exhibited different trends over time. We, therefore, investigated further whether the nature of this opposite trend could be much more relevant than the slope of HR or the slope of MAP taken separately. Therefore, we multiplied HR and MAP slopes to define a new variable. A negative sign suggested that HR and MAP slopes were opposite, whereas a positive sign suggested that HR and MAP followed the same trend (either both increased or both decreased). As a result, Finishers required only two dimensions to explain 96% of the variance. The first dimension (76%) was positively dependent on MAP (coef = 0.95) and the second dimension (20%) was a linear combination of HR (coef = 0.85), grip force offset (coef = 0.38) and age (coef = − 0.34). In Non-finishers, 93% of the variance was obtained using the first two dimensions as well. The first dimension (64%) was again not only positively dependent on MAP (coef = 0.83) but also negatively dependent on HR (coef = − 0.54). The second dimension (29%) was a linear combination of HR (coef = 0.81), MAP (coef = 0.47) and grip force offset (coef = − 0.31). To sum up, this holistic analysis showed that MAP is the most salient variable to predict failure to complete the experiment. This is in agreement with the observation that blood pressure is a primarily related variable during stress application, as was reported first by Julius (Julius 1991) and confirmed in subsequent studies (Goswami et al. 2010, 2011, 2012; Patel et al. 2016).

Beyond absolute MAP, we found that absolute HR (individual raw value, not normalized) was also important when differentiating the two groups. Indeed, we identified HR as a variable in the second dimension in Finishers and as a variable in the first dimension in Non-finishers. Interestingly, the divergent nature of the HR and MAP time series did not allow classification of the two groups into Finishers and Non-finishers. It appears that when categorizing participants into these groups, it is the absolute values of HR and MAP that are more important than whether MAP and HR increase together or change in opposite directions over time.

Understanding mechanisms of cognitive changes and cardiovascular responses induced by AG application, an important countermeasure proposed for missions to, e.g. Moon or Mars, is necessary. Furthermore, our results provide insights into the mechanisms which predispose some persons to presyncope but not others during AG exposure, especially when doing complex tasks. Finally, as the number of mixed sex crewmembers increases, the importance of studies designed to highlight major differences in cognition, handgrip strength and cardiovascular regulation in men and women during space missions also grows.

## Abbreviations

AG: Artificial gravity
ANOVA: Analysis of variance
BMI: Body mass index
Bpm: Beat per minute
GF: Grip force
HR: Heart rate
LF: Load force
MAP: Mean arterial pressure
SEM: Standard error of the mean
